# The role of artificial intelligence in radiotherapy clinical practice

**DOI:** 10.1259/bjro.20230030

**Published:** 2023-10-18

**Authors:** Guillaume Landry, Christopher Kurz, Alberto Traverso

**Affiliations:** 1 Department of Radiation Oncology, LMU University Hospital, LMU Munich, Munich, Germany; 2 German Cancer Consortium (DKTK), partner site Munich, a partnership between DKFZ and LMU University Hospital, Munich, Germany; 3 Bavarian Cancer Research Center (BZKF), Munich, Germany; 4 Department of Radiation Oncology (Maastro Clinic), School for Oncology and Reproduction (GROW), Maastricht University Medical Center, Maastricht, Netherlands; 5 School of Medicine, Vita-Salute San Raffaele University, Milan, Italy

## Abstract

This review article visits the current state of artificial intelligence (AI) in radiotherapy clinical practice. We will discuss how AI has a place in the modern radiotherapy workflow at the level of automatic segmentation and planning, two applications which have seen real-work implementation. A special emphasis will be placed on the role AI can play in online adaptive radiotherapy, such as performed at MR-linacs, where online plan adaptation is a procedure which could benefit from automation to reduce on-couch time for patients. Pseudo-CT generation and AI for motion tracking will be introduced in the scope of online adaptive radiotherapy as well. We further discuss the use of AI for decision-making and response assessment, for example for personalized prescription and treatment selection, risk stratification for outcomes and toxicities, and AI for quantitative imaging and response assessment. Finally, the challenges of generalizability and ethical aspects will be covered. With this, we provide a comprehensive overview of the current and future applications of AI in radiotherapy.

## Background

Machine-learning techniques, widely referred to as artificial intelligence (AI), are supporting automation in several industries including healthcare, and in particular for this review article, in radiation therapy (RT). Techniques such as deep learning (DL), based on convolutional neural networks (CNNs), have found great success in some applications. This is aided by the relatively large volume of annotated imaging data generated in clinical practice in RT, for example organ segmentations on computed tomography (CT) or magnetic resonance (MR) images. While much remains at the research level, some applications, such as automatic segmentation and automatic planning, have begun seeing clinical use. Additionally, new developments in MR-guided RT (MRgRT) at MR-linacs have increased the frequency of online plan adaptation, which brings a greater need for automation and speed, as well as some specific tasks such as converting MR images into pseudo-CT images. Some machine-learning applications in RT have even preceded the recent wave of enthusiasm for AI, such as decision-making and response assessment using AI applied to medical imaging (radiomics), and have contributed to a culture of collecting datasets in RT. Such data collection brings related challenges of generalizibility of AI methods, privacy an ethical concerns. For automated medical image analysis, both machine learning (*e.g.,* radiomics) and deep learning (*e.g.,* deep radiomics) will be impacted by the interobserver variability in the definition of the Regions of Interest (ROIs) from which features are extracted from. Nevertheless, the published literature strongly supports the use of semi-automated methods for the contouring of ROIs. Recent applications in deep learning have bypassed the problem by avoiding ROIs delinations and using bounding boxes around a selected part of the image, or even using as input the whole 3D image. However, this approach may open issues such as: risk of overfitting, introduction of non-relevant parts of the image as input (*i.e.,* noise [background] *vs* signal), and higher computational costs. We strongly suggest, when possible, to benchmark the stability of the results against interobserver variability by collecting data with multiple operators or using publicly available datasets.

**Figure 1.**
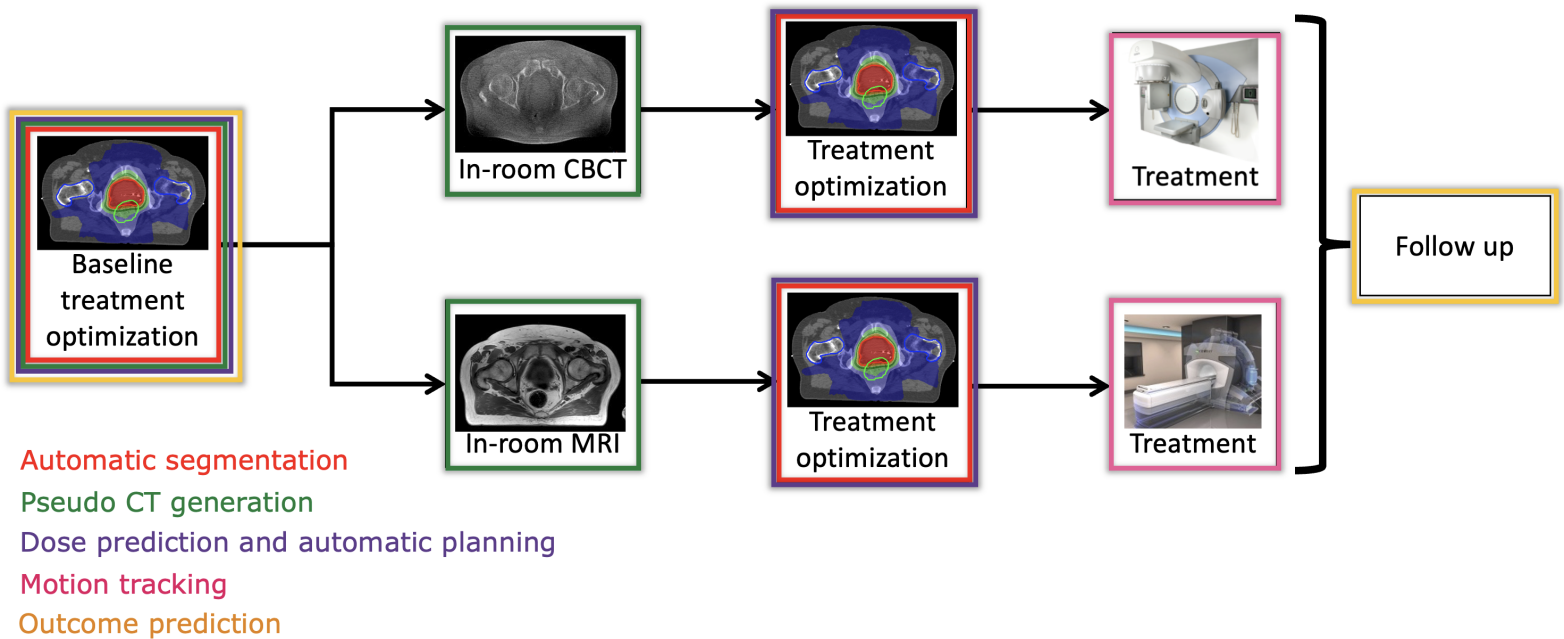
Overview of the modern RT workflow. The top arm represents cone beam computed tomography (CBCT)-guided RT, while the bottom arm represents MRgRT. Various areas of application of AI are highlighted to indicate in which part of the workflow they come into play: automatic segmentation, pseudo-CT generation, dose prediction and automatic planning, motion tracking and outcome prediction.

This review article will cover the current state of AI in clinical practice and provide an overview of potential future applications and challenges. Figure 1 summarizes where AI may play a role in the RT workflow, whether based on cone beam computed tomography (CBCT) image guidance or MRgRT.

## AI in current radiotherapy workflows

The most common application of AI in RT is volume of interest segmentation. For this task, the U-shaped convolutional neural network (U-net) has become the workhorse and consistently produces clinically acceptable results. The method has evolved from 2D segmentation to 3D and has seen the development of dedicated loss functions for model training, such as the Dice similarity coefficient (DSC) or focal loss.^1^ By now, state-of-the-art models come with self-configuration pipelines which determine which hyperparameters are optimal for a segmentation task.^2^ AI auto-segmentation is a reality in several clinics, and multiple vendors now provide commercial solutions for CT images (see for example references^3,4^). Several clinical trials have been initiated to explore auto-segmentation algorithms, *e.g.*, for brain (NCT05093751) or lung lesion segmentation (NCT04164186, NCT05775068). Advantages of automatic segmentation include the reduction of variability among observers^5^ and more importantly time savings in clinical practice. Currently, it is accepted that automatic segmentation is not to be used in an unsupervised manner, but that performing correction of automatic contours saves time compared to a solely manual workflow.^6,7^ Evaluation of the benefits of AI segmentation make use of a set of metrics which include classical geometrical analysis such a DSC and Hausdorff distance, but also expert evaluation using a grading scale.^8^ The geometric metrics do not readily reflect the main clinical advantage, namely, time saving. For this, besides directly recording correction times, metrics such as surface DSC and added path length (APL) may be useful, and Vaassen et al^7^ report that APL correlates the most with time savings.

Another area where AI has made inroads into clinical practice is at the level of automated treatment planning. This part of the workflow will dramatically reduce the time required for an RT technician to deliver a clinically acceptable treatment plan and is thus an area where efforts toward AI automation have been made.^9,10^ One approach based on machine-learning (ML, referring here to models such as support vector machines or random forests) has been knowledge-based planning,^11^ where features such as organ and target distances or number of beams are passed to a ML model predicting characteristics of the dose distribution, such as dose volume histograms (DVH) points. These can then be used in an optimizer as objectives, leading to an automatic procedure. More recently, DL methods, again using U-nets, have been used to predict an entire dose distribution instead of a limited set of DVH points, which allows to steer the optimizer to directly achieve that dose distribution.^12^ Interestingly, in a study where knowledge-based planning was used clinically, the authors reported that 61% of automatic treatment plans were selected. This was surprising since in a simulated environment, a higher percentage of automatic plans (83%) had been selected, indicating that there remain hurdles to the adoption of this technology in clinical routine.

## AI for online adaptive radiotherapy

Online adaptive radiotherapy has been made feasible thanks to the clinical introduction of MR-linacs such as the 0.35 T ViewRay MRidian^13^ or 1.5 T Elekta Unity,^14^ where MR images are used to perform online plan adaptation, typically in a hypo-fractionated setting.^15,16^ In parallel, CBCT-based systems making use of AI, such as the Varian Ethos,^17^ have also recently allowed for clinical online adaptive radiotherapy. Whether CBCT- or MR-based, the online adaptive workflow is one of the areas of radiotherapy with the highest demand for automation. With the patient on the treatment couch, it is highly desirable to accelerate the various steps required to perform plan adaptation, namely, pseudo-CT generation, delineation and plan optimization. At modern MR-linacs, relatively long fraction durations are reported, ranging from 30 min up to 70 min.^18–20^ Delineation can take up to 30 min and currently the main bottle-neck of the online adaptation process.^21^

Several studies have investigated automatic segmentation specific to MRgRT,^5,22–29^ some of them applied clinically.^30^ While most segmentation algorithms employ morphological MRI sequences, also studies making use of diffusion-weighted imaging exist.^31^ This is well summarized in the review by Cusumano et al.^32^ Given the richness of data available, the online adaptive workflow also brings opportunities for novel approaches to automatic segmentation. For example, it may be possible to exploit patient-specific training approaches,^33^ where we assume that high-quality, physician-approved planning contours may be available at the time of plan adaptation for a given fraction. In that case, a conventional baseline model trained on a population dataset could be refined on the planning data of a single patient.^8^ Alternatively, the training could be refined as a function of added fraction images.^34^ Others have trained models from scratch on planning images of an individual patient only,^35,36^ however issues of robustness may arise in this case, especially in the presence of pronounced anatomical changes. In general, when training or fine-tuning models in a patient-specific fashion, care has to be taken to ensure that the model is not overly matched to the planning image, to the detriment of performance on fraction images. One approach is to select a set of patient datasets where fraction images are used as a validation set to monitor the patient-specific training. In this way, it can be ensured that the model performance is optimal on fraction images by selecting an optimal augmentation strategy and training epoch, which will be subsequently fixed for all new patients.^8^ Another issue here is that delineation errors on the planning image may be propagated to the fraction images; however, the availability of more time for planning segmentation should mitigate this. Another way to exploit planning segmentations is to use AI-based DIR, as done in Eppenhof et al for CTV segmentation.^37^ While they achieved convincing results for CTV, limited evidence is available as to the feasibility of this approach for larger deformations for organs such as the bladder or rectum. The studies listed above were focused on MRgRT; however, similar approaches should be applicable to CBCT-based online adaptive RT. In the latter case, however, additional challenges might arise due to the considerably lower soft tissue contrast of CBCT images and the lack of available ground-truth segmentation on clinical CBCT images. In consequence, CBCT segmentation might be combined with domain adaptation to support auto-segmentation.^38^

For pseudo-CT generation, also representing a domain adaptation task, several well-established deep-learning methods have by now been identified either for CBCT to CT or MRI to CT conversion, as well reviewed by Spadea et al^39^ or Gurney-Champion et al for MRI.^40^ The most common approach is to use a U-net, as used by Han et al for MRI to CT^41^ and Kida et al for CBCT to CT.^42^ For this approach, paired data are required. This functions well for relatively static anatomies such as the head and neck or brain, where deformable image registration (DIR) may allow correcting slight anatomical mismatches between CBCT/MR and CT scans.^42^ Alternatively, unpaired training may be used for anatomical sites where DIR may not be sufficient to correct for anatomical variations. For this, generative adversarial networks (GAN) provide an elegant solution.^43^ The cycleGAN architecture in particular allows to use unpaired data, affording flexibility when collecting datasets for network training. Kurz et al applied this to CBCT to CT conversion with success.^44^ A potential drawback of these generative models is that they might suffer from reduced geometric fidelity, such that additional cross-modality loss terms constraining the networks might be required.^45,46^

Another area where AI may play a role in online adaptive MRgRT is for motion tracking on cine-MRI. At the 0.35 T MRIdian MR-linac for example, 2D cine-MRI images are acquired at each fraction for beam gating at a frequency of either 4 Hz or 8 Hz. While gating is a very robust way of ensuring target coverage, it does come at the cost of increased treatment times, with duty cycles as low as 20%.^47^ An alternative which has seen recent interest for MRgRT is multi-leaf collimator (MLC) tumor tracking,^48^ which can be achieved by rigidly or deformably shifting the MLC to match the tumor centroid displacement and shape as observed on 2D cine-MRI. Recent proof-of-principle work at the 1.5 T Unity MR-linac showed that MLC tracking should be feasible,^49,50^ and the Australian MR-linac project has even demonstrated tracking multiple targets.^51^ Optimal MLC tracking requires target tracking^52^ and accounting for system latency, which has been reported to be about 350 ms for different MR-linacs.^51,53,54^ One approach to account for latency is motion prediction, and AI methods such as long-short-term memory (LSTM) networks have been shown to outperform non-AI methods for this.^55,56^ Another interesting approach for motion tracking itself would be again to use DIR networks to deform a key frame to a moving frame.^57^

## Decision-making and response assessment

Advances in RT delivery techniques and the advent of promising new therapies such as immunotherapy or targeted therapies have created a sort of “explosion of treatments”,^58^ from which doctors and patients have to choose. Not only the portfolio of therapies has increased, but many of these treatments interact among each other (*e.g.,* RT enhancing immune response). Personalized medicine refers to choosing the optimal treatment strategy and regimen based on each single patient tumor fingerprints, which are extracted using data mining techniques based on routinely acquired data related to the medical history of the patients, such as electronic health records, genetic testing, and medical imaging.^59^ AI offers a diverse choice of multiple data analytics algorithms that can support personalized medicine.

## Personalized prescription and treatment selection

Many treatment indications in RT (and oncology in general) strongly rely on the results obtained from randomized controlled clinical trials (RCT). If on one hand, RCTs are recognized for their capability of isolating confounding factors during the comparison of treatments (‘control groups’); on the other hand, the “one size does not fit all” is a well-known limitation of RCTs. The upcoming challenge in personalized medicine is to use the knowledge derived from RCTs but adapted on a patient-to-patient basis using routinely collected data during the patient journey: real-world evidence.^60^ AI algorithms can support this practice, given their capability of mining a vast amount of unstructured data and find correlations with an outcome of interest. It is important to point out that the role of AI in personalized prescription and treatment selection is not to replace RCTs, but rather suggesting, for example, one among two treatments that have shown comparable outcomes (*e.g.,* local control, survival) but will provide for example less toxicities. These AI algorithms are often referred to as decision support systems (DSS).^61^ The most optimal DSS will be based on an estimate of the probability of the outcome (*e.g.,* treatment response) *vs* the risk of unwanted toxicities (*e.g.,* RT side-effects). However, it is difficult to use AI to change the dose prescribed to a tumor, given the fact that dose prescriptions are determined by strict clinical guidelines and considering also that the relation among changes in the dose released to a biological tissue and the resulting DNA changes are still under investigation. At the time of writing treatment decisions and clinical constraints on doses to be prescribed are based on the NTCP (Normal Tissue Control Probability) and TCP (Tumor Control Probability) models. However, the research community is investigating how AI can complement and enhance the above models by introducing tumor- and host(organs at risk)-derived fingerprints. An advanced example is the study by Scott et al, where the investigators validated a genomic-adjusted radiation dose, which is a method that accounts for biological heterogeneity and can be used to predict optimal RT dose for an individual patient.^62^ They have shown that using data from the RTOG 0617 trial, 80% of the patients could have been exposed to unnecessary dose escalation without benefit to TCP. A complementary overview of the applications of AI for personalized prescription is provided by the special issue edited by Rancati et al.^63^ The studies vary from the integration of radiosensitivity biomarkers during treatment planning (similarly to Scott et al), but most of the original research studies in the collection focus on the application of AI algorithms for the modeling of toxicity outcomes in clinical cohorts. Cancer sites include brain tumors, head-and-neck, and thoracic diseases (mainly breast cancer, lung, and esophageal cancers), and prostate cancer, and substantial AI algorithm heterogeneity can be found. Some studies prefer to focus on more traditional ML algorithms such as logistic regression or random forest, up to studies that included image-derived biomarkers (radiomics/deep learning) and finally research that uses advanced AI methods such as convolutional neural networks beyond the previously cited U-NET, or even recurrent neural networks in case of time series data (*e.g.,* data acquired during each delivery fraction). It is impossible to draw a conclusion whether more traditional AI algorithms should be preferred over more complex ones, since the choice of the AI algorithms cannot be separated from the type of data that are mined. For example, in case of genomic data, more classical AI algorithms have been chosen compared to when using imaging, dosimetry or clinical data, probably showing that embedding biological knowledge of the interaction of radiation with the tumor/host helps reducing the complexity of the AI models. What emerges is the lack of studies that pool all the possible data available and try to investigate correlations among them. Therefore, we urge the research community to collaborate to provide recommendations for the most optimal choice of the AI algorithms to be used according to the data available.

## Risk-stratification for outcomes and toxicities

As mentioned earlier, AI algorithms can also be used to select the optimal treatment for a patient using DSSs that evaluate the likelihood of treatment response *vs* toxicities. The same DSSs can also be used to isolate groups of patients with different hazard ratios for the risk of an event such as early death, poor response, or the development of metastases. Moving from the identification of these risk groups into an actual actionable for the clinician to be discussed with the patient is not trivial. At the time of writing, many of the AI studies trying to isolate high-risk *vs* low-risk patients can be used for example to propose adjuvant treatment for the high-risk group, while preserving the low-risk group from unnecessary harmful additional radiation. Examples of these are the studies by Wu et al^64^ and Mattonen et al^65^ who looked at identifying early-stage lung cancer patients at higher risk of developing distant metastasis that might benefit from SBRT and/or systemic therapy instead of lobectomy, despite being in good fit for surgery. Another example of AI-based risk stratification is the development of combined imaging/clinical signatures for the identification of small cell lung cancer patients that might benefit from PCI (Prophylactic Cranial Irradiation).^66^ Many studies have also associated risk stratification with treatment response prediction. These AI models can have a strong impact in supporting the selection of patients that will benefit from adjuvant treatment following RT such as immunotherapy. Examples are the studies by Duffy et al^67^ and by Trebeschi et al.^68^ First prospective clinical trials are currently recruiting patients to explore outcome as well as toxicity prediction by means of AI in a clinical setting, *e.g.,* for head and neck cancer (NCT05607225, NCT05081531). It is worth mentioning that almost all the studies investigating risk stratification and treatment response/ toxicity prediction made a strong use of mining imaging data. As already mentioned in the previous sections, RT planning/delivery and monitoring strongly rely on the use of medical images, which come as “cost-free” data to be analyzed. Overall, we are also seeing a trend toward using more and more complex deep learning-based algorithms to mine imaging data.

## Reproducibility, generalizability, and distributed learning

Despite the clinical endpoint investigated, we found that many AI algorithms suffer from the issues of reproducibility and generalizability. Reproducibility refers to the ability of being able to fully reproduce the results of a research study developing an AI model on another dataset, without the need of re-training the algorithm from scratch. Many studies have raised the attention over the lack of reproducibility of AI studies in RT independently from the type of algorithms chosen from the data used and for the outcome of interest.^69,70^ We can mainly distinguish two causes behind this issue: A) poor reporting and lack of standardized good practices (both for data collection and algorithms), and B) complexity of the AI models during its whole lifecycle from development to training and deployment. To solve the first issue, consortia of researchers have been focusing on the development of guidelines that will hopefully support reproducibility of developed AI algorithms.^71,72^ Again, we have noted a lack of agreement among the different consortia, so that it possible only to isolate a small subset of recommendations common among these publications. To solve the second issue, stating that from now on the research community should discard the use of complex AI algorithms, often borrowed from outside the medical field, is not the solution. This is because the choice of the AI algorithms and their complexity with respect to the data types that are mined is still not standardized. Nevertheless, there are methods to overcome the complexity of these algorithms for deployment and make them easily accessible to other researchers or even to clinical professionals. Examples of these are common repository such as GitHub, and modelHub.AI for release of the code, while for the straightforward use of the models, Docker containers should be the preferrable solutions. Another interesting opportunity is to bypass the installation of the software (*i.e.,* an AI algorithm) within one institution and adopt a SaaS (Software as a Service) approach, which is typically achieved by Cloud infrastructures.^73^

Next to reproducibility, the issue of generalizability of AI models, overall and for RT, remain a critical issue impeding a fast clinical translation of these powerful technologies. Generalizability refers to the capability of an AI algorithm to hold prognostic/predictive values or more in general to be robust on heterogenous datasets, besides the data used for training. Generalizability is the opposite of overfitting. Therefore, generalizability strongly relies on the availability of a large amount of annotated data in RT. Annotating and collecting data is expensive, and annotations can vary among different RT clinics because of different clinical guidelines. If solutions like auto-contouring or auto-planning can help in reducing the burden of manually annotating data, still the issue of centrally collecting the data remains. Even in the presence of deep-learning algorithms that can generate synthetic data (*e.g.,* Generative Adversarial Models for pseudo-CT), a large dataset needs to be collected for training the algorithms. Data produced by the use of AI algorithms should be stored and re-used to re-train or fine-tune the algorithms to achieve better performances and eventually increase clinical applicability. The ecosystem produced by AI algorithms, the operators using the algorithms and the results of this interaction (*e.g.,* measured outcome *vs* predicted outcome) should move toward a self-learning healthcare system. Examples of promising applications are self-learning algorithms, or reinforcement learning.^74^ Centrally transferring and collecting the data always come with concerns about privacy and the legal path to approval is much longer than the technical obstacles (almost inexistent) that need to be solved. Therefore, distributed learning as a privacy preserving solution, where the model is trained and transferred to the institutions, rather than the data, is becoming more and more popular, also supported by enterprise research on distributed learning infrastructures. Recent publications have shown not only the possibility to fully develop machine-learning algorithms in a distributed fashion for outcome prediction^75^ but also for the development of distributed deep learning algorithms for auto-contouring^76^ and again prognostication/predictions.^77^ Performance metrics used to evaluate AI algorithms might not be fully representative of the real clinical applicability and value of these algorithms. The literature suggests the use of DCA (Decision Curve Analysis) as an objective quantification of clinical impact. In the case of AI algorithms for segmentation, the usage of reading tests (*e.g.,* Turing test) is strongly recommended to evaluate, for example, the clinical acceptance of automated contours, compared to for example just using a DICE score. Finally, we strongly support the idea of performing clinical trials where the impact of AI algorithms in supporting clinical decisions is evaluated in a controlled setting.

## Ethical challenges

Currently, while many AI algorithms have been proposed, few have become a clinical standard. A first requirement is that prospective evaluation is important to make sure that the results of retrospective evaluation were not biased by data selection and curation. In other words, application to real-world data is needed to determine the performance of an AI algorithm. This is important since some models rely on feature selection by the model designer (for example, radiomics models) with choices made based on the retrospective data used to train the model. This makes these models semi-automatic in nature, and potentially the selected features may not be ideal for prospective data or different populations.

In that context, ethical, medicolegal and quality control aspects are critical. Questions such as the obligation to inform patients on the use of AI tools and of responsibility when AI does harm are far from answered. The frequency of AI quality control evaluations to detect drifts in the input data is also an open question. A good summary of these points can be found in Bhowmik et al.^78^

The interaction of humans with machines and algorithms is a field of active investigations. Introducing AI systems in the consolidated process of decision-making and RT planning/delivery requires a dedicated process of change management. Naik et al stated that the legal and ethical issues that confront society due to AI include privacy and surveillance, bias, or discrimination.^79^ In their analysis, the authors underline how there are no well-defined regulations in place to address the legal and ethical issues that may arise due to the use of AI in a healthcare setting. Without going into detail, we believe that the key questions that we are asked to solve as the RT/oncology community is: does AI fit within the existing legal categories? Should a new category with its special features and implications be developed? Sub-questions related to the applications of AI presented in this review are: 1) “If new data are generated with AI, who is the data owner? Should informed consent be required again? 2) Since AI algorithms are trained on specific collected data, which may have human biases, is AI really supporting equality of care? 3) Is it possible to make decisions based on data that do not have real-world counterparts (*e.g.,* AI pseudo-generated/synthetic data), and 4) Are AI algorithms medical devices and do we need to re-design clinical trials to evaluate and approve AI algorithms? We believe that all these questions demand to be evaluated by not only RT and healthcare professionals but also involving policymakers and governmental agencies.
